# Moringa, Milk Thistle, and Jujube Seed Cold-Pressed Oils: Characteristic Profiles, Thermal Properties, and Oxidative Stability

**DOI:** 10.3390/foods13091402

**Published:** 2024-05-02

**Authors:** Haifa Sebii, Sirine Karra, Abir Mokni Ghribi, Sabine Danthine, Christophe Blecker, Hamadi Attia, Souhail Besbes

**Affiliations:** 1 Laboratory of Analysis Valorization and Food Safety, National Engineering School of Sfax, University of Sfax, Sfax BP:3038, Tunisia; haifasebii@gmail.com (H.S.); karra.sirine@gmail.com (S.K.); mokniabir@yahoo.fr (A.M.G.); hamadi.attia@gmail.com (H.A.); 2 Highly Institute of Biotechnology of Beja, University of Jendouba, Jendouba 9000, BP:382, Tunisia; 3 Laboratory of Food Science and Formulation, Gembloux Agro-Bio Tech, University of Liège, Passage des Déportés 2B, B-5030 Gembloux, Belgiumchristophe.blecker@ulg.ac.be (C.B.); 4 Highly Institute of Applied Sciences of Medenine, University of Gabes, Road El Jorf—Km 22.5, Medenine BP:4119, Tunisia

**Keywords:** vegetable oils, cold press, seeds, quality, thermal, oxidative stability

## Abstract

Cold-pressed moringa, milk thistle, and jujube seed oils were investigated in terms of their characteristic profiles, thermal properties, and oxidative stability. The findings proved that the extracted oils were characterized by high nutritional values, which encourages their use in various fields. Results showed significant differences between the obtained oils. Overall, jujube seed oil exhibited the best quality parameters, with acidity equal to 0.762 versus 1% for the moringa and milk thistle seed oils. Milk thistle seed oil showed absorbance in the UV-C (100–290 nm), UV-B (290–320 nm), and UV-A (320–400 nm) ranges, while the moringa and jujube seed oils showed absorbance only in the UV-B and UV-A ranges. Concerning bioactive compounds, jujube seed oil presented the highest content of polyphenols, which promoted a good scavenging capacity (90% at 10 µg/mL) compared to the moringa and milk thistle seed oils. Assessing the thermal properties of the obtained oils showed the presence of four groups of triglycerides in the moringa and milk thistle seed oils, and two groups of triglycerides in the jujube seed oil. The thermograms were constant at temperatures above 10 °C for milk thistle seed oil, 15 °C for jujube seed oil, and 30 °C for moringa seed oil, which corresponded to complete liquefaction of the oils. The extinction coefficients K_232_ and K_270_, monitored during storage for 60 days at 60 °C, proved that jujube seed oil had the highest polyphenols content and was the most stable against thermal oxidation.

## 1. Introduction

*Moringa oleifera* (L.), *Silybium marianum* (L.), and *Zizyphus lotus* (L.) are considered important medicinal plants that have been used since ancient times. *Moringa oleifera* is native to the Indian subcontinent [1]. *Silybium marianum*, also called “milk thistle”, is native to North Africa and the Mediterranean [2] and is a wild plant that grows abundantly in southern Europe and Asia near waterways. The jujube tree (*Zizyphus lotus*), known in Tunisia under the name of “sedra”, is present in the wild and threatened with extinction. The jujube tree exists in North Africa, from Morocco to Egypt. In Tunisia, moringa is not widespread. Milk thistle and jujube are abundant throughout the territory, but mainly in the south [3]. Since ancient times, different parts of moringa have been used for their well-known healing properties, particularly for infectious diseases and cardiovascular, gastrointestinal, blood, and liver disorders [4]. Milk thistle is mainly used as a medicinal herb, especially for curing liver diseases, due to the presence of active silymarin compounds [5]. The jujube tree has also been used for its antihypertensive, anti-inflammatory, and antimicrobial activity [6]. Over the years, several studies have been interested in establishing relationships between the chemical composition and the biological activities of medicinal plants. Among the parts that have been investigated, the seeds showed an exceptional richness in lipids, present at levels of 41, 26, and 32.9% for moringa, milk thistle, and jujube seeds, respectively [7,8,9]. As a result of this composition, several studies have focused on extracting vegetable oils from the seeds of these plants [2,4,10]. Indeed, vegetable oils are extremely important for human nutrition thanks to their richness in fatty acids and triglycerides, which are important for health. The gap between demand and necessity is widening, mainly due to climate change, which is significantly affecting the harvest of oil-producing crops and thus driving up the price of commercialized oils. 

Multiple methods have been used to extract vegetable oils from different parts of plants, either chemically or mechanically. The main aim is to maximize the yield, but care must also be taken to ensure that the quality of the oil is affected as little as possible. Researchers have used solvents such as methanol, chloroform, petroleum ether, etc. [11]. Mechanical extraction is one of the oldest methods of oil extraction. It consists of reducing the volume occupied by the seeds, while applying pressure, to extract the oil. The advantage of this method is that the oil obtained is of good quality [11]. 

In this context, our study aimed at extracting vegetable oils from moringa, milk thistle, and jujube seeds by cold-pressing, and comparing the obtained oils in terms of their physical properties, quality, biological activity, and oxidative stability. In addition to these properties, the thermal properties were also studied to predict the thermal behavior of the lipids during storage and in different food systems. This information promotes the use of the investigated vegetable oils instead of conventional oils in different fields, such as agri-food, cosmetics, and even pharmaceuticals.

## 2. Materials and Methods

### 2.1. Raw Material

Moringa fruits, collected in September 2022, were supplied from a nursery located in the town of Mornag in Ben Arous, Tunisia. Seeds were manually isolated, and impurities were removed. Milk thistle flowers and jujube fruits were collected from the Medjez El Beb region, Beja Tunisia, in August 2022. Milk thistle flowers were shaken to obtain seeds. For jujube fruits, after washing and drying in the open air, the edible part (pulp) and the stones protecting the seeds were separated manually. Subsequently, the kernels were shelled, allowing the recovery of the seeds. All seeds were washed, air-dried, and kept cool at 4 °C for further use. To ensure representativity, three samples of 10 g of each seed were crushed with a mortar and pestle for physicochemical analysis. This crushing method preserved the quality of the seeds, as it did not generate heat.

### 2.2. Physico-Chemical Analysis

#### 2.2.1. Water Activity

The water activity (aw) was measured at 25 °C using a laboratory aw meter (Novasina, swift aw, Lachen, Switzerland). 

#### 2.2.2. Color

CIE lab parameters of the color space, L*, a*, and b*, were measured using a digital colorimeter (Konica Minolta chromameter CR-5) calibrated with black and white tile.

For the oils, CieLab coordinates (L*, a*, and b*) were directly read with a spectrophotocolorimeter (Tintometer, Lovibond PFX 195 V 3.2, Amesbury, UK). 

L*: brightness, ranging from 0 (black) to 100 (white). 

a*: redness, extending from −100 (green) to +100 (red). 

b*: yellowness, extending from −100 (blue) to +100 (yellow). 

The angles of hue (h°) and chroma or intensity (C*) were calculated according to the following equations:

(1) $$C^{*}=\sqrt{{a*}^{2}+b{*}^{2}}$$

(2) $$h{}^{\circ}=arc\;tang\;\frac{b*}{a*}$$

#### 2.2.3. Refractive Index

The refractive index of the oil samples was determined at 25 °C using a refractometer (ABBE-Kern Optics, Germany) [4].

#### 2.2.4. Density

A known mass of oil was poured into a graduated cylinder. Then, the volume was determined, and the density at 25 °C, expressed in g/mL, was calculated by a simple ratio between the mass and the volume of the oil.

#### 2.2.5. Ultraviolet-Visible Spectrum

The absorbances of the vegetable oil samples were measured at different wavelengths (from 190 to 500 nm) using a spectrophotometer (Shimadzu, Shanghai, China).

#### 2.2.6. Dry Matter

1 g of each of the powdered seed samples was placed at 105 °C into a previously tared crucible until a constant mass was reached (AOAC, 1995) [12].

#### 2.2.7. Ash

To remove carbon, about 1 g of the powdered seed samples was ignited and incinerated in a muffle furnace (L5/11/B180, Nabertherm, Lilienthal, Germany) at 550 °C for about 12 h (AOAC, 1995) [12].

#### 2.2.8. Fat

A mass of 2 g of each type of seed was crushed and introduced into a dry flask. Then, 2 mL of ethanol, 2 mL of formic acid, and 3 mL of HCl (70%) were added to the sample. The mixture was heated to 50 °C for 20 min. After cooling, 4 mL of ethanol and 25 mL of hexane were added. The flask was stirred for 20 min before being centrifuged at 4500 rpm for 20 min. The upper phase was transferred to a dry-tared flask. The hexane was evaporated at 70 °C. Finally, the flask was weighed to determine the fat content [13].

#### 2.2.9. Protein 

From the previous crushed samples, a mass of 250 mg of powdered seed was used to determine total protein content using the Kjeldahl method (AOAC, 1995) [12]. Protein was calculated using a nitrogen conversion factor of 6.25. 

#### 2.2.10. Carbohydrate 

Carbohydrate was estimated by the difference in mean values, i.e., [total solids − (protein + lipids + minerals)] [14].

#### 2.2.11. Phenolic Compounds

20 mL of ethanol (70%) was added to 1 g of each powdered seed. The mixture was stirred in an orbital shaker for 2 h. The separation of solid particles was carried out by centrifugation at 4500 rpm for 20 min, and the supernatant was collected in a dark bottle. The pellet underwent the same extraction under identical conditions. The two supernatants were combined and used for the following experiments [15].

In a test tube, a volume of 2.5 mL of Folin reagent (diluted ×10) was added to 500 µL of the sample. The mixture was stirred by vortex and left to stand in the dark for 8 min. Then, a volume of 2 mL of Na_2_CO_3_ (75 g/L) was added to the mixture, before shaking and kipping in the dark for 15 min [16]. The absorbance at 765 nm was measured. Gallic acid was used to obtain a standard curve, with concentrations varying from 0 to 50 mg/L.

#### 2.2.12. Flavonoids

The determination of flavonoid content started with the addition of 1 mL of distilled water and 150 µL of sodium nitrite (NaNO_2_) (150 g/L) to 250 µL of each extract. The mixed tubes were incubated at room temperature for 6 min. Then, 75 µL of 10% aluminum chloride (AlCl_3_) was added, and the mixture was re-incubated under the same conditions. Finally, 1 mL of NaOH (40 g/L) and 25 µL of distilled water were added, and the absorbance was measured at 510 nm against a reagent blank [17]. The flavonoid content was expressed as quercetin equivalent (EQ)/100 g dry matter DM.

### 2.3. Oil Extraction Procedure

Seeds were cold-pressed using a vegetable oil press (Henan Vic Machinery CD. Ltd., Zhengzhou, China). 

The press had a stainless-steel body (food grade stainless steel) and worked on an electric power supply equal to 1200 w, with a capacity of 10 kg per hour. The used nozzle had a diameter of 25 mm. Extraction conditions were standardized to not exceed 25 °C. A sample of 250 g of each seed was used for oil extraction over 5 min at a speed of 45 rpm. The operation was fully completed after 5 min. Oil extraction was carried out when the seeds were ground and compressed under the pressure exerted by the conical rotation of the screw. The oil was forced into the perforated tube. The cakes were then evacuated at the end through an orifice. The obtained oils went through a centrifugation step to separate the oil from the residual plant debris. Extracted oils were bubbled with nitrogen, stored in vials, and protected against light at 4 °C until the analyses were carried out. The oil yield was the ratio between the weight of the oil extracted and the weight of the seeds to be treated.

(3) $$Yield\;\%=\frac{weight\;of\;extracted\;oil}{weight\;of\;used\;seeds}\times 100$$

### 2.4. Bioactive Compounds of Seed Oil

#### 2.4.1. Phenolic Compounds

For the preparation of the extract, 2.5 g of oil was homogenized with 5 mL of hexane and 5 mL of methanol–water (80:20). Centrifugation was then carried out for 10 min at 5000 rpm, which allowed separation of the two phases. The lower phase, containing phenolic compounds, was recovered.

Then, a volume of 200 μL of the extract was combined with 500 μL of Folin–Ciocalteu reagent, and 4.3 mL of distilled water was added to adjust the volume to 5 mL. Then, 1 mL of Na_2_CO_3_ sodium carbonate solution (30%) and 4 mL of distilled water were added. After mixing, test tubes were incubated for 2 h in the dark at 25° [18]. The absorbance was measured at a wavelength of 726 nm. The content of phenolic compounds was determined according to the following formula:

(4) $$\mathrm{Phenolic}\;\mathrm{compounds}\;(\mathrm{meq}\;\mathrm{AG}/100\;g)=(A_{726}\times \;832.33)+10.26$$

where A_726_ was the absorbance at 726 nm.

#### 2.4.2. Chlorophylls

The determination of the content of chlorophyll pigments in the seed oils was carried out according to the method described by Gutfinger [19]. This method is based on quantification by spectrophotometry at the following wavelengths: 630, 670, and 710 nm. Oils were poured directly into a 1 cm thick glass tank, and the absorbance was read at the wavelengths above. The chlorophyll content was determined by the following formula:

(5) $$Chlorophylls\left(ppm\right)=\frac{\left[A670-\left(\frac{\left(A630+A710\right)}{2}\right)\right]}{\left(0.1086\times L\right)}$$

where A630, A670, and A710 represented the relative absorbances at 630 nm, 670 nm, and 710 nm, respectively, compared to a reference glass containing carbon tetrachloride, and L was the length of the glass (1 cm).

#### 2.4.3. Carotenoids

The determination of the carotene content was based on a spectrophotometric method. A portion of 3 g was introduced into a 10 mL flask, then made up to the mark with cyclohexane and shaken. Next, the absorbance at 470 nm was measured in a glass cell, against cyclohexane as a blank [20]. The carotene content was determined by the following formula:

(6) $$Carotenoїds\left(ppm\right)=\frac{A470\times 25\times 10,000}{2000\times 7.5}$$

where A470 was the absorbance at 470 nm, compared to a reference tank containing cyclohexane.

### 2.5. Antioxidant Activity

The free radical scavenging activity method (DPPH: 2,2-diphenyl-1-picrylhydrazyl) was used to assess the antioxidant activity of the oil extracts [21]. In the reaction tubes, 375 μL of ethanol and 125 μL of DPPH solution were added to 500 μL of the crude extract and dilutions. For each concentration, a blank was prepared by mixing 500 μL of sample and 500 μL of ethanol without adding the DPPH solution. A control tube was also prepared by mixing 500 μL of the solvent (without sample), 375 μL of ethanol, and 125 μL of the DPPH solution. After shaking, tubes were placed in the dark at 25 °C for 1 h. Then, the absorbance values were measured at 517 nm by a spectrophotometer (Shimadzu, UV-VIS Spectrophotometer-UV mini 1240), and the radical scavenging activity was determined using the following formula:

(7) Scavenging activity %=(A Control+A sample−A blank)/A control×100

where A control, A sample, and A blank were the absorbance at 517 nm of the control, sample, and blank tubes, respectively.

### 2.6. Quality Indicators

#### 2.6.1. Acidity

A mass of 2.5 g of oil was added to 10 mL of neutralized 95% ethanol. The titration was carried out with a solution of NaOH (0.1 M) in the presence of phenolphthalein as a colored indicator (ISO 660: 2009). 

#### 2.6.2. Peroxide Value

To a mass of 0.5 g of oil, volumes of 5 mL of chloroform, 7.5 mL of acetic acid, then 0.5 mL of saturated KI solution were added while stirring. The mixture was left to stand in the dark for 10 min. To stop the reaction, 25 mL of distilled water was added in the presence of a few drops of starch as a colored indicator. Then, a titration of the iodine released was carried out using a solution of sodium thiosulfate, Na_2_S_2_O_3_ (0.1 N), until the coloring disappeared. Results were expressed as meq O_2_/kg to AOAC (2000) [22].

#### 2.6.3. Specific Extinction Values

The K_232_ index corresponded to the absorbance of conjugated dienes and their oxidation products. The K_270_ index corresponded to the absorbance of the conjugated trienes and the secondary products of oxidation (carbonyl compounds) [23]. In a 25 mL volumetric flask, 100 mg of oil was added, made up to capacity with the cyclohexane solvent, and homogenized. Then, the sample was poured into a 1 cm thick quartz glass, and the absorbance was read using a UV-visible spectrophotometer at wavelengths of 232 and 270 nm (Shimadzu UVmini-1240, Shanghai, China). 

#### 2.6.4. Saponification Index

Ethanolic potassium hydroxide (0.5 N) was pipetted into conical flasks containing 1.0 g of each sample. The content of each flask was refluxed for 45 min with occasional shaking, then cooled to room temperature, after which it was titrated with sulfuric acid (0.5 N), using phenolphthalein as an indicator. A blank was subjected to the same treatment. Results were expressed as mg KOH/g (ISO 3657: 2013).

#### 2.6.5. Iodine Index

To a mass of 2 g of oil, chloroform was added to reach 100 mL as the solution’s final volume, titrated with Wij’s solution (5 mL), mixed thoroughly, and allowed to stand in the dark for 5 min. Potassium iodide solution (5 mL; 7.5%) was added and titrated to a light straw color using 0.1 N sodium thiosulphate solution. The starch indicator was thereafter added, and titration continued to a colorless endpoint. Results were expressed as gI_2_/100 g (Wij’s method: ISO 3961: 2013).

### 2.7. Fatty Acids Profile

To determine the fatty acid composition of the oil, we opted for the cold transesterification method. A portion of 0.1 g of the oil sample was mixed with 2 mL of heptane and 0.2 mL of methanolic potassium hydroxide solution (2 N) according to AOAC (2000) method 965.33 [22]. The tube was closed and shaken vigorously for 30 s. After standing, the upper part of the solution, which contained the methyl esters, cleared following its decantation. The heptane solution was thus ready for injection into the GC. Analyses of fatty acid methyl ester (FAME) were carried out using an AGILENT 8860 Series (G2790A) gas chromatograph (AGILENT technologies, Waldbronn, Germany) equipped with a hydrogen flame ionization detector (AGILENT) and a capillary column (50 m × 0.25 mm × 0.20 µm Agilent, Waldbronn, Germany). The column temperature was programmed from 180 to 220 °C at 5 °C/min and the injector and detector temperatures were set at 240 °C. Identification and quantification of FAME were accomplished by comparing the retention times of peaks with those of pure standards purchased from Sigma-Aldrich (Sigma Chemical Co., Sofia, Bulgaria) and analyzed using the same conditions. The results were expressed as a percentage of individual fatty acids in the lipid fraction.

### 2.8. Thermal Profile

The thermal properties of the samples were determined using differential scanning calorimetry (DSC) performed using a Q1000 DSC thermal analysis instrument (TA DSC Q1000, New Castle, DE, USA) with a cooling accessory. A sample test portion of approximately 10 ± 2 mg was encapsulated in an airtight aluminum capsule. The heating and cooling cycles were carried out under a flow of nitrogen (50 mL/min). The device was calibrated at a heating rate of 5 °C/min and cooling rate of 10 °C/min between temperatures of −40 °C and 40 °C [24]. Heating and cooling thermograms were obtained using “Universal Analysis” software version 4.5 (TA instruments, Newcastle, DE, USA). The temperature values provided information about the temperature at which the melting process began, the temperature at which most of the triglycerides had melted, and the complete melting temperature of the oil.

### 2.9. Oxidative Stability

A mass of each extracted oil sample was placed in closed bottles in an oven thermostatically controlled at 60 °C (under accelerated oxidation conditions). The specific extinction coefficients K_232_ and K_270_ were monitored during storage. Two samples of each set were removed from the oven every 15 days for triplicate analysis [25]. At the end of the storage period, fatty acid profiles and polyphenol contents were assessed.

### 2.10. Statistical Analysis

The results obtained in the current study were analyzed using SPSS 20 software. Means with a significant difference were determined by applying Duncan’s multiple range test at a significance level (*p* < 0.05). All analytical determinations were performed at least in triplicate. Values were expressed as the mean ± standard deviation.

## 3. Results

### 3.1. Physico-Chemical Characterization of Seeds

Table 1 summarizes the results of all of the physical and chemical properties of the seeds. The seeds of *M. oleifera* (MOS), *S. marianum* (SMS), and *Z. lotus* (ZLS) were characterized by low humidity, in the order of 6.79, 5.51, and 7.18%, respectively. The results were similar to those obtained in the studies of Leone [26] for moringa, lower than that reported by Anum, Raja [2] for milk thistle seeds, and similar to those obtained in studies of Abdeddaim, Betka [27] and Chouaibi, Mahfoudhi [9] for jujube seeds. The chemical composition of the seeds showed that fat was the main constituent of MOS and SMS; it represented 36.39 and 27.99% of the dry mass of the seed for moringa and milk thistle, respectively. The obtained results showed that the two seeds had different chemical compositions (*p* < 0.05), particularly for proteins. Indeed, apart from the oil, protein content was around 31.33 and 17.19% for moringa and milk thistle, respectively. The composition of ZLS was almost equally divided between three main components, which were proteins (32%), carbohydrates (30%), and lipids (26%). The protein content was higher than that provided by Chouaibi, Mahfoudhi [9] (19%) and Abdoul-Azize [28] (14%). The fat contents of the studied seeds exceeded percentages reported for prickly pear seed (8%), but were less than those of sesame (52%) and argan seeds (53%). MOS had the same fat content as nigella seeds (37%) [29]. 

In terms of bioactive molecules, results showed that the polyphenol content of SMS (403.91 meq GA/100 g) was the highest of the studied seeds, followed by MOS (359.23 meq GA/100 g), and finally ZLS (310.04 meq GA/100 g). Other studies have shown that polyphenols existed more abundantly in the leaf and fruit parts of the jujube tree, at 664 and 4078.2 mg/100 g, respectively [30,31]. On the other hand, the flavonoid content of moringa seeds (0.6 mg EQ/100 g) was lower than that of jujube seeds (1.09 mg EQ/100 g) and that of milk thistle seeds (1.24 mg EQ/100 g). 

### 3.2. Characteristic Profiles of Cold-Pressed Seed Oils

#### 3.2.1. Physical Properties

Concerning the appearance of the extracted oils, compared to *S. marianum* oil (SMO) and *Z. lotus* oil (ZLO), *M. oleifera* oil (MOO) had the highest values of L* and b*, and the lowest value of a*, implying a bright green-yellowish color. SMO was characterized by the strongest red color, and ZLO had the smallest yellow tint (Table 2). 

The observed density of moringa seed oil (0.904 g/mL) was in agreement with what was reported by Tsaknis [1], but was higher than that of milk thistle seed oil (0.820 g/mL) and of jujube seed oil (0.860 g/mL). The refractive index (RI) of oil is related to the degree of saturation [32]. The extracted oils had comparable RI values. They had values similar to the literature for MOO [33], for SMO [34], and for ZLO [35].

**Figure 1:**
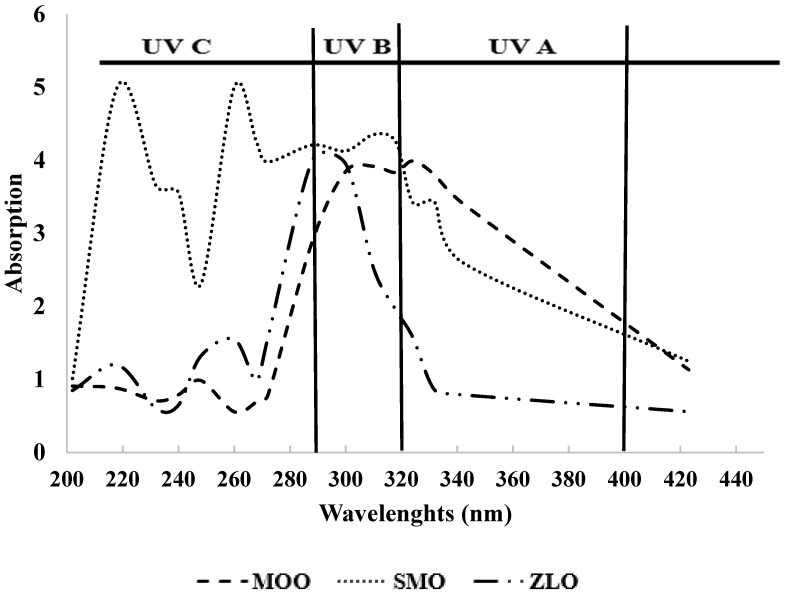
UV absorption profiles of moringa, milk thistle, and jujube seed oils.

The absorbance of SMO (Figure 1) showed intense absorption peaks between 190 and 240 nm. Figure 1 shows that MOO had majority peaks around 300 nm, with a very weak shoulder between 190 and 250 nm. MOO and SMO showed peaks at 290 and 340 nm, corresponding to a carbonyl transition [36]. SMO showed absorbances in UV-C (100–290 nm), UV-B (290–320 nm), and UV-A (320–400 nm) ranges, while MOO and ZLO showed absorbance only in the UV-B and UV-A ranges. The UV spectra of date seed oil showed that Allig seed oil had some absorbance in the UV-C, UV-B, and UV-A ranges [14], which is similar to that observed for SMO. The optical transmission of MOO, SMO, and ZLO, especially in the UV range (290, 400 nm), was comparable to that of titanium dioxide preparations with a sun protection factor for UV-B (SPF) and a protection factor for UV-A (PFA) [37]. 

#### 3.2.2. Quality Indicators

The extraction yield was 17% for MOO and ZLO, and 14% for SMO (Table 2). Thus, around 50% of the initial fat was extracted from *M. oleifera* and *S. marianum* seeds, and 65% from *Z. lotus* seeds. Tsaknis [1] reported a better extraction yield of cold-extracted moringa seed oil (25.8%). The yield of SMO obtained in our study was lower than that found by El-Haak, Atta [38] via solvent extraction (29%). El Hachimi and El Antari [10] reported a better oil extraction yield for jujube (29%) compared to pomegranate and prickly pear, whose yields were around 23 and 8%, respectively.

The acidity values (Table 2), expressed as a percentage of oleic acid per 100 g of oil, showed that ZLO had the best quality among the extracted vegetable oils, given that it had the lowest acidity value (0.753%), which was also lower than that of *Z. lotus* seed oil from Morocco, extracted by the Soxhlet method (1.22%) [10], and similar to that of prickly pear seed oil (0.71%) [29]. However, the recorded value was higher than that of argan oil (0.28%) [29]. MOO presented a value of 1.03%, which was in agreement with that obtained by Anwar, Ashraf [39], and greater than the value reported by Campas-Baypoli, Sánchez-Machado [40]. SMO showed almost the same acidity as MOO in this study. All acidity values were lower than that of nigella seed oil (2.3%) [41]. MOO and ZLO in this study had a K_232_ value of around 1, and K_270_ values equal to 0.02 and 0.12, respectively, while SMO had a K_232_ value of around 2 and a K_270_ value equal to 0.30. SMO also had the highest recorded peroxide value (2.57 meq O_2_/kg), compared to MOO and ZLO.

The saponification index (IS) is the mass of potassium hydroxide (KOH), expressed in milligrams, necessary to neutralize the free fatty acids and saponify esterified fatty acids contained in one gram of fat. MOO and SMO possessed IS values equal to 189 and 192 mg KOH/g, respectively, while ZLO had the lowest IS value (182 mg KOH/g). This proves the presence of free fatty acids in the obtained oils. Findings were consistent with those reported for *M. oleifera* seed oil of Malaysian origin [42], for *S. marianum* seed oil [34], and with that found by Chouaibi, Rezig [35] for *Z. lotus* seed oil. The iodine value of moringa seed oil (52.7 g I_2_/100 g) was lower than the values declared for *M. oleifera* oils from Mexico, Malaysia, India, Nigeria, and Pakistan, ranging from 66.2 to 85.3 g I_2_/100 g of oil [40,42]. The obtained result for SMO (66.7 g I_2_/100 g) was lower than the value obtained by Fadhil, Ahmed [34] (99 g I_2_/100 g). As for ZLO, the recorded value (90 g I_2_/100 g) was similar to that reported by Chouaibi, Mahfoudhi [9], who reported that jujube seed oil had the lowest iodine value among olive, corn, soybean, sunflower, groundnut, jujube seed, and rapeseed oils. 

#### 3.2.3. Bioactive Compounds and Antioxidant Activity

The bioactive compound (polyphenols, carotenoids, and chlorophylls) contents of the oils were determined immediately after their preparation (Table 2). MOO had the highest contents of carotenoids and chlorophyll, attaining 16.36 and 5.85 ppm, respectively, which explained its greenish color. The obtained values were lower than those reported in the literature for the same oil [43], which could be explained by the effects of storage, or by the used extraction method. Our values were in agreement with those reported for the Jaffa variety from India [44], with 16.9 ppm of carotenoids. The carotenoid and chlorophyll contents of MOO also exceeded those recorded for prickly pear seeds, reaching a maximum of 3.68 and 3.33 ppm, respectively [30]. On the other hand, we noted that SMO was not as rich in pigment as MOO, having 8.21 and 0.24 ppm of carotenoids and chlorophyll, respectively. Kachel and Krajewska [45] reported that milk thistle seed oil possesses chlorophyll and carotenoid contents of approximately 2.69 and 49.85 ppm, respectively, which was higher than the results of our study. The lowest values of chlorophyll and carotenoids were noted for ZLO, reaching only 1.27 and 0.08 ppm of carotene and chlorophyll, respectively. 

Polyphenols were present in significant amounts of 425.3, 105.42, and 94.04 ppm for ZLO, SMO, and MOO, respectively. The values obtained are of great significance, as they are much higher than the total phenolic content observed in extracts of Opuntia ficus indica, which have ranged from 4.8 (red variety) to 8.9 ppm (orange variety) [46]. The polyphenol content of ZLO also exceeded the values seen in virgin olive oils, which have varied between 110.6 and 350.4 ppm [47]. Several studies have established correlations between polyphenol content and antioxidant activity [48]. It is therefore necessary to investigate the antioxidant activity of the oils. The DPPH radical neutralization test is one of the most commonly used tests to study antioxidant activity. At the same concentration (10 μg/mL), the oils showed very interesting antioxidant activity, reaching 90, 68, and 59% for ZLO, SMO, and MOO, respectively (Table 2). The demonstrated anti-radical activity in this study was greater than that reported by Ogbunugafor, Eneh [4], who discovered activity equal to 50% at 50 µg/mL for moringa seed oil (of Nigerian origin), and exceeded that of Javeed, Ahmed [49] (76% at a concentration of 50 μg/mL for SMO). Moreover, Snoussi, Koubaier [3] showed that *Z. lotus* seed oil collected from different locations in Tunisia exhibited antioxidant activity against DPPH reaching 50% for concentrations ranging between 4.08 and 6.63 mg/mL (expressed in terms of IC50), which is much lower than the obtained result for ZLO in our study. 

#### 3.2.4. Fatty Acid Profile

The composition of fatty acids is a decisive parameter that makes it possible to assess the quality of an oil. Analysis by gas chromatography provided the fatty acid compositions of the oils, shown in Table 3. In general, there was a clear predominance of unsaturated fatty acids (USFA) compared to saturated fatty acids (SFA) for MOO, SMO, and ZLO, with proportions of 80.7, 79.7, and 85% USFA, respectively, and 19, 21, and 15% SFA, respectively. Monounsaturated fatty acids (73, 25, and 65%) predominated compared to polyunsaturated fatty acids (7.4, 54.6, and 20%).

The main saturated fatty acids in MOO were palmitic, stearic, and behenic acids, although oleic acid was the main unsaturated fatty acid (73.2%), with small amounts of palmitoleic, linoleic, and linolenic acids. The obtained results for MOO were similar to those presented by Lalas and Tskanis [50]. For SMO, three saturated fatty acids were identified: palmitic, stearic, and arachidic acids. Linoleic acid was the most abundant unsaturated fatty acid in SMO. The noted percentages were similar to those reported for cottonseed oil, which was found to have contents equal to 19% and 52% of oleic and linoleic acids, respectively [51]. Different results have been reported by Hasanlou and Bahmani [52] for milk thistle seed oil.

ZLO had five major fatty acids, namely oleic, linoleic, palmitic, stearic, and gadoleic acids. The latter, which is a monounsaturated omega-9 fatty acid primarily found in different vegetable oils such as olive oil, peanut oil, and canola oil, presents several health benefits, including increasing HDL cholesterol levels and reducing inflammation in the body, which is associated with various chronic diseases such as arthritis, cardiovascular disease, and metabolic syndrome. These findings were similar to those presented by El Hachimi, El Antari [10] for the same type of oil. Another study carried out on the fatty acid profile of pistachio mastic oil showed a ratio (USFA/SFA) similar to that of oils extracted from jujube seeds [35]. Like the ZLO obtained in this study, the FAs of virgin olive oil are divided into 86% USFA and 14% SFA [53]. All studied vegetable oils exhibited trans fatty acids (C18:1T) and (C18:2T + C18:3T) contents of less than 0.05% [54]. 

Jujube seed oil is an unsaturated-type oil and could be classified as oleic-linoleic oil, with the predominance of oleic over linoleic acid, contrarily to milk thistle seed oil, a linoleic-oleic oil, characterized by the predominance of linoleic over oleic acid. While *M. oleifera* seed oil belongs to the category of oleic acid oils [55], it had approximately the same oleic acid content as olive oil (72.21%), but a much lower linoleic acid content (11.8%) [50]. 

#### 3.2.5. Thermal Profile

**Figure 2:**
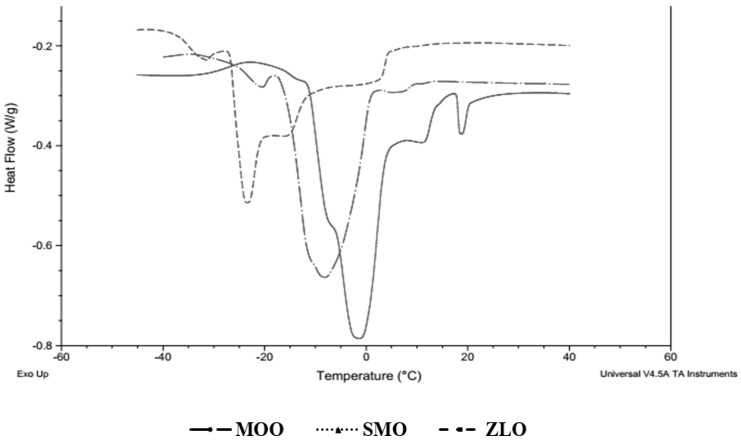
Thermal profiles of moringa, milk thistle, and jujube seed oils.

The thermograms showed endothermic peaks, proving that the MOO, SMO, and ZLO samples absorbed energy that allowed them to pass from the crystalline to the liquid state, corresponding to a sudden melting phase during heating in the DSC. The thermal profiles showed that there were major peaks and minor peaks (Figure 2). The results of the thermal analysis of the oils, presented in Table 4, showed that MOO had a melting peak at a temperature of −1.57 °C and a melting enthalpy of 74.01 J/g, different from those of SMO (−23.47 °C and 58.82 J/g) and those of ZLO (−7.90 °C and 62.47 J/g). The melting field that occurred between the initial and the final temperatures showed a superposition of peaks related to the melting phenomena of the different TAG families. The peaks obtained were asymmetric and could indicate the presence of four TAG groups for MOO and SMO and two TAG groups for ZLO, which have different weights with different melting points [56]. The initial and final temperatures of all of the oil melting processes were also variable, and the transition temperatures for the main peaks of the three samples were different. The thermograms were constant at temperatures above 10 °C for SMO, 15 °C for ZLO, and 30 °C for MOO, which corresponded to complete liquefaction of the oils. 

#### 3.2.6. Oxidative Stability 

**Figure 3:**
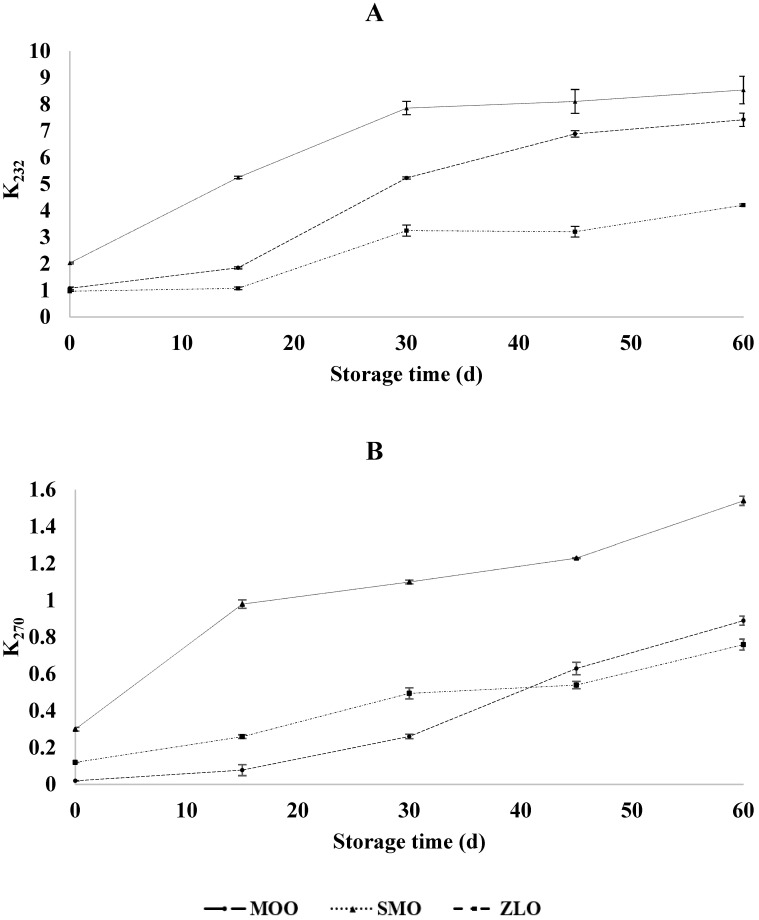
Effect of storage for 60 days at 60 °C on oxidative indicators of moringa, milk thistle, and jujube seed oils: K_232_ (**A**) and K_270_ (**B**).

The experiment carried out allowed us to evaluate changes in the extinction coefficients during the oxidation of the vegetable oils throughout 60 days of storage at 60 °C. Figure 3 shows that the specific extinction coefficients increased during storage at 60 °C, which is related to the fact that the temperature accelerated the oxidation of fatty acids, which occurs without the heating effect. This oxidation was less important for ZLO than for MOO and SMO, as the values after 60 days at 60 °C were 4.2 and 0.76 for K_232_ and K_270_, respectively, for ZLO, while the values for MOO and SMO were higher, namely 7.42 and 8.54 for K_232_ and 0.89 and 1.54 for K_270_.

After oxidation, polyphenol content was assessed, and the results showed an important decrease in the content of these compounds. Table 5 showed that almost 24% of polyphenols were conserved for ZLO, against 21% for MOO, and 15% for SMO, after storage. 

To confirm this fact, the FA composition of moringa, milk thistle, and jujube seed oils after storage at 60 °C is shown in Table 5. After storage, variations in the contents of different FAs were recorded. Total USFA and SFA were affected significantly (*p* < 0.05) by thermal oxidation. USFA decreased notably, while SFA increased for all oil samples. The ratio of C18:2/C16:0 can be used to indicate the degree of oxidative degradation of the oil, as this ratio decreases after oxidation. From the reported values (Table 5), we can observe the same evolution of this ratio for all oil samples during storage at 60 °C, which decreased to 0.24, 2.11, and 1.45, after initially being 0.41, 5.8, and 1.83 for MOO, SMO, and ZLO, respectively. The same trend was recorded for refined olive oil stored for 60 days at 60 °C, which was attributed to its thermal oxidation [25]. 

## 4. Discussion

The determination of the physicochemical properties was carried out to gain a precise understanding of the different components of the seeds. Indeed, characterization makes it possible to choose the optimal methods of using these seeds. The humidity value, with a relatively low water activity value, ensures better storage of seeds and reduces the risk of microbial contamination and lipid peroxidation. Differences in lipid and protein contents could be attributed to plant varieties, soil type, growing climates, and harvest seasons. From the chemical composition, it can be deduced that all seeds were characterized by a high nutritional value and were of exceptional interest due to their remarkable protein and lipid contents. They can thus constitute an interesting matrix for the extraction of vegetable oil.

Cold-pressed extraction gave rise to three vegetable oils: *M. oleifera*, *S. marianum*, and *Z. lotus* seed oils (MOO, SMO, and ZLO). Characteristic profiles, quality indicators, and the thermal stability of the obtained oils were assessed to promote their use in different fields. Overall, all of the vegetable oils were of an acceptable bright color, which encourages their use in food formulations. Concerning yield, around 50% of the initial fat was extracted from *M. oleifera* and *S. marianum* seeds, and around 65% from *Z. lotus* seeds. The moisture content of the seed affected the yield of oil. It appeared that low moisture content leads to lower oil yield, which was inferred from the recorded values for milk thistle seeds, with the lowest moisture content and the lowest value for oil yield. 

The presence of absorption in the ultraviolet range constitutes useful information to better understand the structure of the different components of the oils and indicates the absence of saturated lipids, alkanes, aliphatic amino acids, and sugars in the soluble fractions. The absorbance of SMO proved that this oil could contain unsaturated carbonyl groups and carboxyl groups. The observed decrease in absorption in the visible region (400–500 nm) can be explained by the presence of colorless (transparent) compounds in the oil composition. It should be noted that in the UV-B and UV-A ranges, wavelengths are responsible for most cellular damage. Thus, the extracted oils are capable of protecting against UV-B and UV-A rays, and can be used in the formulation of anti-UV protectants. The information provided by this analysis can be used to inform the choice of the field of application of the obtained oils; for example, they could be used in cosmetic creams, given their potential to have an effect against ultraviolet radiation.

The determination of quality criteria was necessary to evaluate the degree of oxidation of oils. Among quality indicators, acidity reflects the degree of hydrolysis of an oil. ZLO had the greater quality, since extra virgin olive oil has a free acidity equal to a maximum of 0.8% [54]. Both the MOO and SMO oils were also of good quality, given that virgin olive oil has a free acidity of less than 2% [54]. The extinction coefficients at 232 and 270 nm of a fatty substance, like the peroxide index, are also indicative of the oxidative deterioration of oils. Indeed, the oxidation of oil leads to the formation of primary oxidation products, which absorb light around 232 nm. As oxidation continues, secondary products form and absorb light near 270 nm. These parameters reflect the good quality of MOO and ZLO. For SMO, these results showed that SMO oil contains primary (hydroperoxides) and secondary oxidation products. Low acidity, as well as the extinction coefficients at 232 and 270 nm and peroxide values, indicate that existing triglycerides did not exhibit significant oxidation and proved that the oils are edible and might have a long shelf life [35,56], since obtained values remained inferior to that of virgin olive oil [54].

The analysis of the iodine and saponification indices was necessary to characterize the oils and determine the storage conditions. Thus, the differences observed in these indices could be the cause of differences in the oxidative stability, physical state, and thermal behavior of these oils. The extraction methods and the origin of the plants had no influence on the saponification index. It should be noted that a low degree of unsaturation indicates that an oil is less sensitive to the phenomenon of lipid peroxidation. In fact, the lower iodine values in this study indicated the good stability of the studied oils, which was related to the lower degree of unsaturation of the constitutive fatty acids. 

Polyphenols, carotenoids, and chlorophylls are natural antioxidants that have a protective action against peroxidation. Carotenoids are conjugated terpene molecules, which explains their strong absorption in the visible range (470 nm). The presence of such bioactive molecules helps protect oils against rancidity and gives them a special flavor. The protective effect of carotenoids and chlorophylls is ensured through light absorption, which degrades the chlorophylls, causing a loss in the oil color. Polyphenols are molecules well known for their activities in neutralizing free radicals and protecting against oxidative stress. In addition, they can provide benefits to human health and prevent cardiovascular and carcinogenic diseases [57].

From the observed bioactive molecule values, SMO was the most susceptible to lipid oxidation, while ZLO was able to resist this phenomenon better. The differences could be attributed to the origin of the seeds or the degree of oxidation. The obtained results demonstrated a very significant correlation between the concentration of polyphenols and the capacity to trap free radicals, as demonstrated by the literature [58,59]. It should also be noted that although the highest recorded values for chlorophyll and carotenoids were found in MOO, this type of oil showed the least scavenging activity, which can probably be attributed to the pro-oxidant effect of these natural molecules. Findings proved that oils obtained by cold-pressing from *S. marianum*, *M. oleifera*, and *Z. lotus* seeds could present excellent antioxidant properties during storage and in various food, pharmaceutical, and even cosmetic formulations. 

The low content of trans fatty acids proved that the oils obtained are of good quality and do not present a risk to the health of consumers. The high oleic acid content in MOO makes it desirable in terms of nutrition and cooking, and more stable when frying. On the other hand, Lecerf [60] showed in his study that highly monounsaturated oils have low fluidity, and when heated, their stability will be greater compared to other oils rich in polyunsaturated fatty acids, which is the case of the oils obtained in this work. The dietary importance of these oils is based on their high composition of unsaturated fatty acids, especially oleic acid, which are known for their positive impact in opposing the activity of free radicals. *M. oleifera* seed oil can be used as a natural source of behenic acid, which has been used as an oil-structuring and solidifying agent in margarine and foods containing semi-solid and solid fats, eliminating the need to hydrogenate the oil [61].

Differential scanning calorimetry (DSC) is a rapid and direct way to evaluate the thermal behavior of cold-pressed extracted oils during heating and cooling. Using this method, phase transition temperatures and enthalpies can be determined. The peak superposition may be due to the inter-solubility of the TAGs constituting the oils, or the phenomenon of lipid polymorphism generating new forms of TAG. Given that the melting point depends on the FAs constituting the TAGs (length of the carbon chain and saturation), as well as their position on the corresponding alcohol, the melting of polyunsaturated TAGs occurred at lower temperatures [56,62]. The lower melting temperature was observed for milk thistle seed oil, showing a higher content of PUFA (53.37%) compared to jujube seed oil (19.74%) and moringa seed oil. (5.92%). The information provided by this part of the study could be useful for fractionation tests of TAG families with different fusion domains, which can be applied in the agri-food field as a techno-functional ingredient.

Monitoring heat stability is an important step before using vegetable oils in various fields including agri-food, cosmetics, and pharmaceuticals. During the oxidation of oils, the formation of primary products leads to an increase in light absorption at 232 nm. As oxidation progresses, secondary products are formed that absorb light at 270 nm. Oxidation stability is improved by synergistic interactions between various natural antioxidants in the oil itself. This stability allows an initial assessment of the shelf life of an oil [63]. Thus, K_232_ and K_270_, are efficient indicators for the conjugation of trienes and the presence of carbonyl compounds, respectively [64]. As mentioned in the section regarding bioactive compounds, ZLO, which had a higher resistance to thermal oxidation, had the highest content of polyphenols, which play a major role in protecting oil from lipidic peroxidation phenomena. On the other hand, Ayadi and Grati-Kamoun [64] reported that the maximum permitted values for K_232_ and K_270_ are, respectively, 2.50 and 0.20 for extra virgin olive oil, and 2.60 and 0.25 for virgin olive oil. From this, we may conclude that MOO and ZLO remain edible until 15 days at 60 °C, as K_232_ and K_270_ reached only 1.85 and 0.078 for MOO and 1.08 and 0.26 for ZLO, respectively. On the other hand, SMO exceeded the critical threshold before 15 days. The highest recorded values for SMO could also be explained by the initial values at the beginning of the experiment, which could be due to the initial quality of the *S. marianum* seeds. Anum, Raja [2] reported that the selection of a good collection period, in the case of *S. marianum*, is difficult because flowering continues throughout its life cycle. Therefore, the seeds used in this study appeared to have already changed in their plants before harvest.

The oxidation phenomenon also affects the nutritional value of food, as it leads to the loss of essential vitamins and amino acids, as well as essential fatty acids. According to Zribi and Gargouri [65], linoleic and palmitic acids are generally the most used indicators of the degree of deterioration of fatty substances, because linoleic acid is the most sensitive to oxidation, while palmitic acid is more resistant to oxidation. The results confirmed that ZLO, whose C18:2/C16:0 ratio decreased the least, was the most resistant to thermal oxidation, followed by MOO and SMO. The latter, which was the most susceptible to oxidation, exhibited the greatest decrease in the C18:2/C16:0 ratio. Considering the high applied temperature (60 °C), the studied oils could have a good shelf-life and thus be safely stored over a relatively long period of time. This hypothesis should be supported by monitoring the quality indicators during storage at room temperature.

## 5. Conclusions

This analysis of cold-pressed oils from moringa, milk thistle, and jujube seeds proved that they can be used as an alternative vegetable oil source. The preliminary study showed that the extracted oils had a desirable bright color and that they could absorb UV radiation. The established comparison showed that the greatest differences were observed in the bioactive compounds, with the oil from jujube seeds having the highest polyphenols content, which resulted in the best scavenging activity, compared to the moringa and milk thistle seed oils. These latter oils exhibited antioxidant activity that was due, in addition to polyphenols, to their carotenoid and chlorophyll contents. Fatty acid profiling showed that milk thistle seed oil was the most polyunsaturated oil, which caused it to melt at lower temperatures. However, this oil was found to be the most sensitive to thermal oxidation, compared to jujube seed oil, which resisted the lipid peroxidation phenomena better. Finally, the obtained thermal properties provide useful information for further fractionation experiments to promote the use of the studied oils in different fields. All results obtained proved that the extracted vegetable oils are of high nutritional quality, and that they can be used as ingredients in food, agriculture, and even cosmetics. Further studies are warranted to demonstrate the influence of the refining process on the heat stability of the studied vegetable oils.

## Figures and Tables

**Table 1:** Physicochemical characteristics of *Moringa oleifera*, *Silybium marianum*, and *Ziziphus lotus* seeds.

Parameters	MOS	SMS	ZLS
aw	0.568 ± 0.001 c	0.511 ± 0.04 a	0.535 ± 0.001 b
Dry matter (%)	93.21 ± 0.28 b	94.49 ± 0.12 c	92.82 ± 0.57 a
Total proteins (% DM)	31.33 ± 0.92 b	17.19 ± 0.69 a	32.17 ± 0.61 c
Lipids (% DM)	36.39 ± 2.8 c	27.99 ± 2.36 b	26.66 ± 0.10 a
Ash (% DM)	3.21 ± 0.07 b	4.69 ± 0.17 c	3.06 ± 0.06 a
Carbohydrates (% DM)	29.07 ± 0.25 a	50.13 ± 0.58 c	38.11 ± 0.61 b
Total phenols (mg GA/100 g DM)	359.23 ± 1.94 b	403.91 ± 25.25 c	310.04 ± 2.15 a
Flavonoids (mg EQ/100 g DM)	0.604 ± 0.02 a	1.24 ± 0.01 c	1.09 ± 0.02 b
Color parameters: Cie Lab			
L*	83.04 ± 0.14 c	38.575 ± 3.04 a	44.16 ± 0.02 b
a*	2.27 ± 0.21 a	4.285 ± 0.09 b	6.27 ± 0.01 c
b*	16.25 ± 1.62 c	8.86 ± 0.63 a	10.63 ± 0.00 b
C*	16.40 ± 1.64 c	13.11 ± 0.97 b	12.35 ± 0.0 a
h°	82.10 ± 0.38 b	65.95 ± 3.65 a	59.48 ± 0.06

MOS: *M. oleifera* seeds; SMS: *S. marianum* seeds; ZLS: *Z. lotus* seeds. Different letters (a,b,c) in the same line indicate significant differences (*p* ≤ 0.05).

**Table 2:** Extraction yields and quality profiles of *Moringa oleifera*, *Silybium marianum*, and *Ziziphus lotus* seed oils.

Parameters	MOO	SMO	ZLO
Yield (g/100 g seed)	17.27 ± 0.45 b	14.39 ± 1.12 a	17.66 ± 0.32 b
Yield (g/100 g Fat)	47.45 ± 0.51 a	51.41 ± 0.98 b	66.24 ± 0.47 c
Color parameters: Cie lab			
L*	52.01 ± 0.77 c	43.28 ± 0.16 b	38.41 ± 0.03 a
a*	1.70 ± 0.27 a	5.23 ± 0.02 c	4.09 ± 0.1 b
b*	70.69 ± 0.74 c	47.71 ± 0.21 b	22.88 ± 0.23 a
C*	70.93 ± 0.72 c	47.74 ± 0.22 b	23.24 ± 0.24 a
H°	85.28 ± 0.00 b	87.95 ± 0.01 c	79.87 ± 0.18 a
Density (g/mL)	0.904 ± 0.02 c	0.820 ± 0.02 b	0.860 ± 0.02 a
Refraction Index	1.469 ± 0.005 a	1.471 ± 0.003 a	1.470 ± 0.002 a
Acidity (%)	1.03 ± 0.06 b	1.08 ± 0.02 b	0.762 ± 0.03 a
K_232_	1.08 ± 0.08 b	2.03 ± 0.10 c	0.97 ± 0.01 a
K_270_	0.02 ± 0.002 a	0.30 ± 0.03 c	0.12 ± 0.00 b
Peroxide Value (Meq O_2_/Kg)	1.48 ± 0.05 b	2.57 ± 0.13 c	0.98 ± 0.05 a
Iodine Index (g I_2_/100 g)	52.7 ± 2.68 a	66.7 ± 4.48 b	90.98 ± 1.45 c
Saponification Index (mgKOH/g)	189.33 ± 0.93 b	192.84 ± 6.94 c	182.51 ± 4.48 a
Polyphenols (ppm)	94.04 ± 9.16 a	105.42 ± 8.89 b	425.3 ± 7.90 c
Carotenoids (ppm)	16.36 ± 0.07 c	8.21 ± 0.41 b	1.24 ± 0.02 a
Chlorophyll (ppm)	5.85 ± 0.01 c	0.24 ± 0.04 b	0.12 ± 0.01 a
Scavenging Activity (%) *	59.98 ± 2.00 a	68.14 ± 1.09 b	90.90 ± 0.45 c

MOO: *M. oleifera* seed oil; SMO: *S. marianum* seed oil; ZLO: *Z. lotus* seed oil. * DPPH tested at a concentration equal to 10 µg/mL. Different letters (a,b,c) in the same line indicate significant differences (*p* ≤ 0.05).

**Table 3:** Fatty acid profiles of *Moringa oleifera*, *Silybium marianum*, and *Ziziphus lotus* seed oils.

Percentage (%)	MOO	SMO	ZLO
Myristic acid C14:0	0.01 ± 0.002 a	0.09 ± 0.006 b	0.09 ± 0.056 b
Palmitic acid C16:0	5.92 ± 0.018 a	9.04 ± 0.04 b	10.04 ± 0.07 c
Heptadecanoic acid C17:0	0.06 ± 0.002 b	0.08 ± 0.001 c	0.05 ± 0.001 a
Stearic acid C18:0	5.22 ± 0.015 b	5.82 ± 0.07 c	4.56 ± 0.05 a
Arachidic acid C20:0	0.67 ± 0.001 b	3.39 ± 0.05 c	0.25 ± 0.02 a
Behenic acid C22:0	6.27 ± 0.02 c	2.3 ± 0.04 a	1.3 ± 0.06 b
Lignoceric acid C24:0	1.12 ± 0.003 c	0.60 ± 0.003 b	0.56 ± 0.002 a
Palmitoleic acid C16:1	1.36 ± 0.004 c	0.08 ± 0.004 a	0.17 ± 0.002 b
Heptadecenoic acid C17:1	0.04 ± 0.001 b	0.03 ± 0.001 a	0.05 ± 0.001 c
Oleic acid C18:1	73.29 ± 5.80 c	25.06 ± 0.07 a	61.06 ± 0.09 b
Linoleic acid C18:2	2.44 ± 0.01 a	52.52 ± 0.02 c	18.41 ± 0.02 b
Linolenic acid C18:3	3.48 ± 0.01 c	0.85 ± 0.1 a	1.33 ± 0.1 b
Gadoleic acid C20:1	0.08 ± 0.003 a	0.15 ± 0.01 b	3.66 ± 0.01 c
C18:1T Trans oleic acid+	0.02 ± 0 b	0.01 ± 0 a	0.01 ± 0 a
C18:2T Trans linoleic acid+ C18:3T Trans linolenic acid	0.02 ± 0 b	0.01 ± 0 a	0.02 ± 0 b

MOO: *M. oleifera* seed oil; SMO: *S. marianum* seed oil; ZLO: *Z. lotus* seed oil. Different letters (a,b,c) in the same line indicate significant differences (*p* ≤ 0.05).

**Table 4:** Thermal profile parameters of *Moringa oleifera*, *Silybium marianum*, and *Ziziphus lotus* seed oils.

Parameters	MOO	SMO	ZLO
Transition temperature (°C)			
T_peak 1_ (°C)	−7.81 ± 0.17 a	−31.90 ± 0.09 c	−21.02 ± 0.2 b
T_peak 2_ (°C)	−1.63 ± 0.17 a	−23.58 ± 0.10 c	−8.1 ± 0.70 b
T_peak 3_ (°C)	11.39 ± 0.07 a	−15.08 ± 0.22 b	-
T_peak 4_ (°C)	18.71 ± 0.12 b	2.82 ± 0.15 a	-
Onset temperature (°C)	−11.72 ± 0.26 a	−27.11 ± 0.08 c	−15.78 ± 0.07 b
Offset temperature (°C)	23.8 ± 0.40 c	9.06 ± 0.25 a	11.46 ± 0.25 b
Peak (°C)	−1.63 ± 0.17 a	−23.58 ± 0.10 c	−8.1 ± 0.70 b
Melting enthalpy ${∆H}_{f}$ (j/g)	74.01 ± 2.42 c	58.82 ± 1.02 a	62.47 ± 012 b

MOO: *M. oleifera* seed oil; SMO: *S. marianum* seed oil; ZLO: *Z. lotus* seed oil. Different letters (a,b,c) in the same line indicate significant differences (*p* ≤ 0.05).

**Table 5:** Effect of storage for 60 days at 60 °C on fatty acid profiles and polyphenol contents of *Moringa oleifera*, *Silybium marianum*, and *Ziziphus lotus* seed oils.

	MOO		SMO		ZLO	
Storage Time (d)	0	60	0	60	0	60
SFA (%)	19.27 ± 0.2 a	22.55 ± 2.2 b	21.32 ± 1.8 a	25.25 ± 1.2 b	14.92 ± 1.8 a	16.22 ± 2.3 b
USFA (%)	80.73 ± 4.70 b	72.58 ± 2.8 a	79.71 ± 3.15 b	71.52 ± 3.45 a	85.08 ± 3.15 b	79.58 ± 1.20 a
C18:2/C16:0	0.41 ± 0.01 b	0.24 ± 0.01 a	5.80 ± 0.01 b	2.11 ± 0.02 a	1.83 ± 0.01 b	1.45 ± 0.01 a
Polyphenols (ppm)	94.04 ± 9.16 b	20.23 ± 1.2 a	105.22 ± 8.89 b	15.87 ± 2.4 a	425.3 ± 7.90 b	99.25 ± 3.1 a

MOO: *M. oleifera* seed oil; SMO: *S. marianum* seed oil; ZLO: *Z. lotus* seed oil. Different letters (a,b,c) in the same line within the same type of oil indicate significant differences (*p* ≤ 0.05).

## Data Availability

The original contributions presented in the study are included in the article, further inquiries can be directed to the corresponding author.
